# Manifesto alimentar: o dilema higiene-segurança face às aflatoxinas com enfoque em Moçambique

**DOI:** 10.1590/0102-311XPT205125

**Published:** 2026-10-26

**Authors:** Edgar Cambaza

**Affiliations:** 1 Universidade Aberta ISCED, Beira, Moçambique.

**Keywords:** Aflatoxinas, Segurança Alimentar, Dilemas Morais, Saúde Pública, Aflatoxins, Food Security, Moral Dilemmas, Public Health, Aflatoxinas, Seguridad Alimentaria, Dilemas Morales, Salud Pública

## Abstract

O presente ensaio examina o dilema ético envolvido na gestão de alimentos contaminados por aflatoxinas, considerando-se a insegurança alimentar em Moçambique, com atenção particular à tensão entre a necessidade de preservar a disponibilidade imediata de alimentos e o dever de proteger a saúde pública. O objetivo foi discutir, à luz da ética normativa, qual a decisão mais justificável em situações de crise: eliminar os alimentos contaminados, transformá-los, desviá-los para usos não alimentares ou admitir o seu consumo sob risco. Trata-se de um ensaio teórico apoiado em análise documental de fontes acadêmicas e institucionais sobre aflatoxinas, segurança alimentar, saúde pública e regulação, tendo Moçambique como foco principal e o Quênia como caso contrastante. A leitura do material foi orientada por perspectivas deontológica, da virtude e consequencialista. Os documentos analisados sugerem que nenhuma resposta está isenta de custos morais em cenários de escassez extrema. A destruição de alimentos pode reduzir o risco sanitário, mas tende a agravar a fome. O consumo sob risco, por sua vez, pode assegurar a sobrevivência imediata, embora exponha sobretudo os grupos mais vulneráveis a danos graves e duradouros. A transformação controlada e o desvio para usos não alimentares aparecem como saídas intermédias, ainda que dependam de capacidade técnica, de regulação efetiva e de alguma transparência institucional. Diante disso, o problema parece exigir uma normatização clara, flexível e participativa, acompanhada de prevenção pós-colheita, comunicação de risco e monitoramento, de modo a reduzir a exposição sem ignorar o direito humano à alimentação adequada.

## Abstract (en)

This essay examines the ethical dilemma involved in managing food contaminated by aflatoxins, considering food insecurity in Mozambique, with particular attention to the tension between the need to preserve immediate food availability and the duty to protect public health. The aim was to discuss, in light of normative ethics, which decision is most justifiable in crisis situations: destroying contaminated food, transforming it, diverting it to non-food uses, or allowing its consumption under risk. This is a theoretical essay supported by documentary analysis of academic and institutional sources on aflatoxins, food safety, public health, and regulation, with Mozambique as the main focus and Kenya as a contrasting case. The reading of the material was guided by deontological, virtue ethics, and consequentialist perspectives. The documents analyzed suggest that no response is free from moral costs in scenarios of extreme scarcity. Destroying food may reduce health risk but tends to worsen hunger. Conversely, consumption under risk may ensure immediate survival, although it exposes especially the most vulnerable groups to severe and lasting harm. Controlled transformation and diversion to non-food uses appear as intermediate solutions, even though they depend on technical capacity, effective regulation, and some degree of institutional transparency. Thus, the issue requires clear, flexible, and participatory regulation, accompanied by post-harvest prevention, risk communication, and monitoring, in order to reduce exposure without ignoring the human right to adequate food.

## Abstract (es)

Este ensayo examina el dilema ético que implica el manejo de alimentos con contaminación por aflatoxinas al considerar la inseguridad alimentaria en Mozambique, con especial atención a la tensión entre la necesidad de preservar la disponibilidad inmediata de alimentos y el deber de proteger la salud pública. El objetivo fue discutir, a la luz de la ética normativa, la decisión más justificable ante situaciones de crisis: eliminar los alimentos con contaminación, transformarlos, desviarlos para usos no alimentarios o admitir su consumo en riesgo. Este es un ensayo teórico respaldado por el análisis documental de fuentes académicas e institucionales sobre aflatoxinas, seguridad alimentaria, salud pública y regulación, con Mozambique como foco principal y Kenia como caso contrastante. La lectura del material estuvo guiada por perspectivas deontológicas, de virtud y consecuencialista. Los documentos analizados sugieren que ninguna respuesta está exenta de costos morales en escenarios de extrema escasez. La destrucción de alimentos puede reducir el riesgo para la salud, pero tiende a empeorar el hambre. El consumo en riesgo, a su vez, puede garantizar la supervivencia inmediata, aunque expone principalmente a los grupos más vulnerables a daños graves y duraderos. La transformación controlada y el desvío a usos no alimentarios aparecen como soluciones intermedias, aunque dependen de la capacidad técnica, de la regulación efectiva y de cierta transparencia institucional.Ante esto, el problema parece requerir una regulación clara, flexible y participativa, acompañada de prevención poscosecha, comunicación de riesgos y monitoreo, para reducir la exposición sin ignorar el derecho humano a una alimentación adecuada.

## Introdução

O dilema das aflatoxinas em contextos de insegurança alimentar assenta no seu duplo impacto na saúde pública e na disponibilidade de alimentos ^1^. As aflatoxinas, metabólitos secundários produzidos por fungos do gênero *Aspergillus*, classificados como carcinogênicos humanos do Grupo 1, contaminam culturas de alto rendimento como milho e amendoim ^2^. A exposição crônica está associada ao câncer do fígado ^3^. Em carência alimentar, atuam como antinutricionais e imunossupressoras, agravando a desnutrição infantil ^4^. O problema é agravado porque famílias em insegurança alimentar, sobretudo agricultores de subsistência, consomem alimentos contaminados em vez de eliminá-los, ampliando a exposição e os riscos ^5^. A contaminação generalizada compromete a segurança alimentar ^6^.

Moçambique no período pós-independência e Quênia em 2004 ilustram respostas contrastantes ao problema. Segundo Casadei ^7^, a escassez alimentar levou Moçambique a restringir a destruição de alimentos contaminados. O autor acrescentou que produtos com níveis de aflatoxinas considerados inaceitáveis eram destinados a operações de transformação (como limpeza, diluição com lotes seguros ou refinação para a produção de óleo) para reduzir a contaminação e preservar o abastecimento alimentar. Em contraste, o surto de 2004 no Quênia, com 317 casos e 125 mortes ^8^, demonstra como a falha no controle resultou numa exposição aguda e fatal ^9^. O milho local apresentava contaminação muito acima do limite regulatório de 20ppb e, ao entrar no mercado, sustentou a exposição generalizada e a elevada mortalidade ^8^. A inclusão do caso do Quênia de 2004 e da obra de Casadei ^7^ não decorre de um critério estrito de atualidade, mas do valor histórico e analítico que ambos conservam. O surto queniano permanece uma referência importante na literatura africana sobre aflatoxicose aguda, e Casadei registra uma resposta institucional moçambicana relevante, baseada na diluição, limpeza ou transformação de produtos contaminados. Em conjunto, essas fontes ajudam a reconstituir antecedentes centrais do dilema entre proteger a saúde e preservar a disponibilidade alimentar.

Cambaza & Koseki ^10^ argumentaram que o dilema entre destruir ou consumir alimentos contaminados reflete a tensão do Objetivo de Desenvolvimento Sustentável 2 (ODS 2, Fome Zero), pois a destruição, embora por vezes necessária, fragiliza o combate à fome ao comprometer a segurança alimentar. Todavia, essa opção compromete o ODS 3 (Saúde e Bem-Estar), uma vez que a exposição crônica às aflatoxinas constitui um problema crítico de saúde pública, associado à elevação da morbidade e da mortalidade ^11^. O problema em causa não se limita ao plano técnico ou regulatório. Ele parece traduzir um conflito moral entre o dever de proteger a saúde, a obrigação de evitar a fome e as exigências de justiça perante populações estruturalmente vulneráveis. Nessa perspectiva, o dilema das aflatoxinas pode ser compreendido como uma tensão entre direitos, consequências e responsabilidades práticas, o que justifica a sua análise à luz da ética normativa.

No quadro legal, Moçambique definiu limites máximos toleráveis para aflatoxinas em determinadas culturas, bem como regulamentos de segurança alimentar e de defesa do consumidor ^4^ ^,^ ^12^. Em condições ideais, esses instrumentos permitem a produtores, comerciantes e decisores prevenir a contaminação e restringir a circulação de alimentos afetados. Contudo, em contextos marcados por tempestades tropicais, conflitos armados e outras calamidades ^13^, as perdas quantitativas e qualitativas das colheitas tornam-se frequentes, obrigando os decisores a privilegiar, em função da gravidade da crise, ora a segurança alimentar, ora a higiene.

Diante desse quadro, o presente ensaio propõe-se a analisar, com base na ética normativa, o dilema entre eliminar, reaproveitar ou consumir alimentos contaminados por aflatoxinas em contextos de crise alimentar em Moçambique. Para isso, consideram-se as implicações de cada decisão nas perspectivas deontológica, da virtude e consequencialista, procurando contribuir para decisões mais informadas e ajustadas à problemática.

## Metodologia

O presente trabalho assume a forma de um ensaio teórico-analítico, apoiado em análise documental orientada pela ética normativa. Trata-se de uma reflexão estruturada com base em documentos acadêmicos e institucionais considerados relevantes para compreender o problema das aflatoxinas em cenários de insegurança alimentar. Note-se que a análise não corresponde a uma revisão sistemática nem apresenta dados empíricos originais.

A análise incidiu sobre publicações em português e em inglês identificadas no Google Scholar (https://scholar.google.com.br/) a partir de 1^o^ de junho de 2025, recorrendo a combinações dos termos “aflatoxinas”, “alimentos”, “Moçambique”, “ética” e equivalentes em inglês. Foram considerados os documentos com enfoque em aflatoxinas, segurança alimentar, saúde pública, regulação e gestão de risco, desde que apresentassem pertinência direta para Moçambique ou, no caso do Quênia, utilidade comparativa. A escolha de uma única base pareceu adequada ao caráter exploratório do ensaio e à necessidade de reunir, num mesmo espaço de busca, a literatura acadêmica e os documentos institucionais. Ainda assim, essa opção pode ter deixado de fora textos melhor indexados em bases mais especializadas, o que constitui uma limitação do estudo.

O Quênia foi incluído como caso contrastante por concentrar episódios agudos de aflatoxicose amplamente documentados, com elevada mortalidade e respostas institucionais que diferem, em pontos importantes, das historicamente observadas em Moçambique. Não se pretendia estabelecer uma comparação epidemiológica estrita. O interesse foi sobretudo analítico: observar como contextos distintos e decisões públicas diferentes podem tornar mais visível o dilema entre proteger a saúde e preservar a disponibilidade alimentar.

Os documentos selecionados foram organizados no Atlas.ti Scientific Software (http://atlasti.com/). Nesse processo, o software teve função sobretudo analítica e organizacional, ao facilitar a reunião e a classificação de excertos relevantes segundo os eixos de discussão do artigo. A codificação não procurou gerar teoria nem categorias emergentes. Serviu, de forma mais modesta, para sistematizar passagens consideradas pertinentes à interpretação normativa.

A operacionalização ética apoiou-se em três perguntas orientadoras aplicadas tanto aos documentos quanto às opções de decisão identificadas. Na perspectiva deontológica, observou-se quais deveres, direitos e normas apareciam afirmados ou violados. Na ética da virtude, a atenção recaiu sobre as disposições morais e as responsabilidades práticas exigidas dos decisores. Já na perspectiva consequencialista, procurou-se examinar benefícios, danos e *trade-offs* associados a cada curso de ação. Esses eixos, ainda que não esgotem a complexidade do problema, ajudaram a orientar a leitura dos textos e a interpretação do dilema central.

O Quadro 1 reúne os principais documentos mobilizados na construção do argumento. A seleção foi intencional e necessariamente limitada, o que parece refletir tanto a escassez de literatura especificamente centrada no caso moçambicano quanto a própria natureza ensaística do estudo. O quadro não esgota o universo consultado, mas destaca as fontes mais diretamente relevantes para a análise.

**Quadro 1:** Principais documentos consultados na presente pesquisa.

FONTES	TÓPICO PRINCIPAL	RELEVÂNCIA PARA A ANÁLISE
Azziz-Baumgartner et al. ^9^	Surto de aflatoxicose no Quênia (2004)	Exemplo de decisão oposta (destruição) e suas implicações
Cambaza et al. ^14^	Revisão sobre aflatoxinas em Moçambique	Síntese histórica e científica da investigação nacional
Cardwell et al. ^37^	Introdução do biocontrole Aflasafe em Moçambique	Alternativa prática à destruição de alimentos
Martinho et al. ^15^	Contaminação de farinhas de milho em Moçambique	Evidência empírica recente sobre exposição local
Matusse et al. ^19^	Aflatoxinas em alimentos básicos (Angola e Moçambique)	Panorama comparativo e dados regionais aplicáveis
Tufts University et al. ^17^	Aflatoxinas, desnutrição e desenvolvimento infantil	Conexão direta entre exposição e saúde pública em Moçambique
Partnership for Aflatoxin Control in Africa ^26^	Estratégias continentais de controle	Enquadramento político africano que inclui Moçambique
Southern African Development Community ^38^	Diretrizes regionais de segurança alimentar	Normas regionais de referência para regulação e harmonização
Sineque et al. ^12^	Ocorrência de aflatoxinas em Moçambique	Diagnóstico sobre prevalência e desafios de controle
Tamele et al. ^16^	Necessidade de monitoria nacional	Argumenta pela criação de sistemas oficiais de vigilância

Entre as limitações mais evidentes, destacamos o uso de uma única base de busca, a heterogeneidade do material incluído e a dependência de literatura secundária para a parte da reconstrução histórica e normativa.

## Fatos relevantes

Esta seção reúne os principais elementos factuais identificados nos documentos analisados e mobilizados como base para a discussão ética subsequente. Não se trata de resultados empíricos originais, mas de achados documentais relevantes para compreender as condições concretas em que o dilema se coloca.

Milho e amendoim são as principais fontes de exposição em Moçambique ^14^ ^,^ ^15^ ^,^ ^16^ ^,^ ^17^ ^,^ ^18^. O milho e seus derivados apresentam maior incidência e níveis de aflatoxinas, particularmente na província de Gaza ^19^. De acordo com Matusse et al. ^19^, o arroz e o sorgo também são motivos de preocupação. Tufts University et al. ^17^ mencionaram a mandioca, mas a evidência em que se basearam é antiga, circunstancial e não conclusiva.

A ingestão de altas doses pode levar à aflatoxicose aguda, com sintomas como vômitos, dor abdominal, icterícia e hepatite grave, podendo resultar em insuficiência hepática fulminante e morte (mortalidade de 25-40% dos casos) ^8^ ^,^ ^20^. A exposição crônica em níveis reduzidos é um forte fator de risco para o carcinoma hepatocelular (câncer do fígado) ^20^, especialmente quando há uma infeção concomitante pelo vírus da hepatite B ^4^. Além disso, as aflatoxinas são imunosupressoras e estão associadas à desnutrição crônica infantil ^1^ ^,^ ^17^.

Além dos efeitos sobre a saúde, as aflatoxinas têm impacto profundo no rendimento dos agricultores e na economia nacional. Segundo a Parceria para o Controle da Aflatoxina em África (PACA, Programa da Comissão da União Africana) ^6^, para os agricultores, a contaminação leva à perda de rendimento devido à rejeição das colheitas ou à redução do valor de mercado. Isso limita a sua capacidade de acessar os mercados mais rentáveis, incluindo o internacional e, consequentemente, reduz a sua capacidade de comprar alimentos para as suas famílias. A desnutrição acarreta custos estimados em aproximadamente 19,9% do produto interno bruto (PIB), devido à má nutrição e à perda de produtividade ^21^.

O dilema é evidenciado em exemplos históricos. No Quênia, o dilema manifestou-se quando houve surtos de aflatoxicose ^20^. Em 1980, 12 pessoas morreram e, de 2000 a 2008, foram registadas 256 mortes, 124 das quais em 2004, causadas por elevados níveis de aflatoxina no milho comercial ^5^. Esses surtos evidenciam o risco de morte associado à exposição a alimentos contaminados. Em Moçambique, o problema surgiu historicamente após uma pesquisa sul-africana sobre o câncer que incluiu famílias de mineiros que retornaram daquele país ^14^. Entre 1968 e 1974, estudos na província de Inhambane detectaram uma incidência excepcionalmente alta de carcinoma hepatocelular, particularmente em adolescentes e jovens, associada à exposição alimentar à aflatoxina ^7^. Mais tarde, a vertente econômica foi realçada pela rejeição, pela União Europeia, do amendoim moçambicano entre 2007 e 2009, por exceder os limites regulamentares de aflatoxina ^20^.

A problemática das aflatoxinas é mais severa nos países em desenvolvimento, com escassez de recursos e de capacidade analítica para o controle ^12^. Em Moçambique, a evidência local disponível é limitada e esporádica. Um estudo em Nampula confirmou a exposição generalizada, com níveis detectáveis no soro sanguíneo de 90% das crianças ^20^. Além disso, altos níveis de contaminação no milho em Gaza e em Inhambane excedem os limites internacionais recomendados pelo *Codex Alimentarius* ^19^. Por outro lado, a insegurança alimentar é comparável e provavelmente mais séria, sendo crônica para aproximadamente 24% das famílias, 27% nas zonas rurais e aguda para, em média, 450 mil habitantes ^22^ ^,^ ^23^. A insegurança alimentar aumenta os custos de saúde; a alimentação representa 48% da despesa das famílias ^23^. Assim, qualquer decisão entre descartar e consumir alimentos contaminados carrega o agravamento de um conjunto de problemas sanitários e socioeconômicos.

## Partes interessadas

No Governo, o Secretariado Técnico de Segurança Alimentar e Nutricional (SETSAN) e o Conselho Nacional (CONSAN) desempenham funções cruciais de coordenação política e técnica da agenda de segurança alimentar ^23^. Os ministérios setoriais, como o da Saúde (MISAU) e o da Agricultura e Desenvolvimento Rural (MADER), são essenciais na implementação, sendo que a saúde e a agricultura representam mais de 70% do orçamento global para a segurança alimentar ^22^. Segundo o SETSAN ^23^, os agricultores, em grande parte pequenos produtores de subsistência, são a base da produção de culturas suscetíveis à aflatoxina (milho, amendoim) e são considerados grupos sociais vulneráveis e titulares de direito, cujas práticas influenciam a contaminação. A organização acrescenta que os comerciantes e o setor privado são parceiros fundamentais na produção, transporte e comercialização de alimentos, tendo a responsabilidade legal de informar o consumidor sobre a nocividade ou periculosidade dos produtos. Os consumidores são os que têm o direito à proteção da saúde e segurança, e são os mais expostos aos riscos de saúde da aflatoxina (como o câncer do fígado ou a desnutrição crônica) ^24^. Por fim, os profissionais de saúde (incluindo o MISAU e o Instituto Nacional de Saúde) são vitais no monitoramento da exposição à aflatoxina, na prevenção de doenças associadas à insegurança alimentar e nutricional e na prestação de cuidados ^23^.

A PACA, a FAO (Organização das Nações Unidas para a Alimentação e a Agricultura) e a OMS (Organização Mundial da Saúde) oferecem enquadramento técnico-político; a Comunidade de Desenvolvimento da África Austral (SADC, acrônimo em inglês) apoia a harmonização e o comércio ^6^ ^,^ ^25^. A PACA apoia a análise e o planejamento de ações (C-SAAP − *Country-led Aflatoxin and Food Safety Situation Analysis*) e gera dados abrangentes para informar decisões sobre políticas e regulamentos ^24^ ^,^ ^26^. A FAO e a OMS promovem programas internacionais de monitoria de contaminantes nos alimentos ^7^ e fornecem apoio técnico e financeiro para o controle da segurança alimentar ^23^. A OMS também fornece referências cruciais sobre padrões de saúde pública ^20^ ^,^ ^27^. A SADC é identificada como uma entidade de comércio regional para cereais, da qual Moçambique participa, sendo também um ator internacional influente no desenvolvimento de políticas alimentares ^22^ ^,^ ^23^. O seu papel é influenciar a aprovação e a implementação de políticas e, em alguns casos, fornecer apoio financeiro ^22^.

A literatura sobre micotoxinas é baixa fora dos meios acadêmicos; a exposição agrava-se com dietas pouco diversificadas; educação e extensão rural são cruciais ^12^ ^,^ ^16^ ^,^ ^28^. O Estado deve garantir o direito humano à alimentação adequada (DHAA), regular a segurança e informar; o setor privado responde pela qualidade e informação ao consumidor; consumidores têm direito à proteção e reparação ^21^ ^,^ ^23^ ^,^ ^24^. Esses deveres, porém, não se distribuem de forma simétrica entre os atores. O Estado concentra poder regulatório e responsabilidade de proteção; o setor privado controla parte importante da circulação e da qualidade dos produtos; os agricultores familiares operam sob forte restrição material; e os consumidores, embora titulares de direitos, tendem a suportar os riscos mais graves com menor capacidade de escolha. Essa assimetria de poder é central para a análise ética, pois permite identificar quem decide, quem se beneficia e quem absorve os custos sanitários e sociais de cada curso de ação.

## Potenciais violações da ética

O DHAA exige simultaneamente quantidade e qualidade, frequentemente com conflito em crise ^4^ ^,^ ^21^ ^,^ ^23^. O consumo de alimentos contaminados, mesmo que satisfaça a necessidade imediata de saciedade, compromete o direito à proteção da saúde (Artigo 92º da Constituição, de forma geral) ^29^, uma vez que a exposição a aflatoxinas representa um risco significativo para a saúde pública, associado ao câncer hepático e à desnutrição crônica ^19^. A contradição reside na escolha ética entre garantir a quantidade (evitar a fome imediata) e garantir a qualidade (evitar doenças crônicas), sendo a flexibilização das normas um dilema que sacrifica a saúde dos mais vulneráveis em prol da sobrevivência ^25^ ^,^ ^30^.

O quadro legal prevê sanções administrativas, civis e penais para produtos perigosos, incluindo apreensão e inutilização ^7^ ^,^ ^31^ ^,^ ^32^. Medidas como limpeza, mistura ou diluição permanecem, contudo, eticamente ambíguas. Sob uma perspectiva deontológica, podem colidir com normas de segurança e com o dever de proteção do consumidor. Numa perspectiva consequencialista, podem parecer compreensíveis em situações-limite, quando a alternativa imediata é a fome. Já sob a ótica da ética da virtude, o problema parece expor falhas de prudência, justiça e responsabilidade pública quando o risco é transferido aos grupos mais vulneráveis. A presença de fungo, a incerteza quanto à redução efetiva da contaminação e a possibilidade de gerar uma aparência enganosa de segurança tornam essas alternativas particularmente controversas ^19^ ^,^ ^21^ ^,^ ^23^.

## Possíveis cursos de ação

A análise das opções apresentadas no Quadro 2 revela que a problemática da gestão de alimentos contaminados por aflatoxinas em contextos de crise ultrapassa a simples escolha binária entre preservação e eliminação. O Quadro 2 sintetiza quatro cursos de ação: destruição total, diluição ou transformação, usos não alimentares e consumo sob risco.

**Quadro 2:** Opções éticas e logísticas para a gestão de alimentos contaminados por aflatoxinas em Moçambique.

OPÇÃO	VANTAGENS	DESVANTAGENS	EM CRISES
Destruição total	Elimina totalmente o risco à saúde; cumpre sanções legais	Muito dispendiosa; agrava a fome e a escassez	Pouco viável: em crises, a sobrevivência prevalece sobre os regulamentos
Diluição ou transformação	Reduz a contaminação sem eliminar todo o lote; aproveita os alimentos	Pode violar normas sanitárias; eficácia limitada; mais viável em armazéns do que em casas	Adotada no passado (guerra/seca) para preservar a quantidade em detrimento da qualidade
Usos não alimentares	Evita o consumo humano direto; pode gerar valor (p.ex.: rações)	Pouco prático; risco à saúde animal e redução da proteína disponível	Baixa prioridade, pois a fome leva ao uso direto para consumo humano
Consumo sob risco	Garante sobrevivência imediata; resposta prática à fome extrema	Risco grave de saúde (câncer, atraso no crescimento, desnutrição)	Realidade inevitável em famílias financeiramente desfavorecidas sem alternativas seguras

Fontes: African Union’s Interafrican Bureau for Animal Resources ^5^, Partnership for Aflatoxin Controlin Africa ^6^, Cambaza et al. ^10^, Ezekiel et al. ^11^ e Tufts University et al. ^17^.

Nenhuma opção é universal. A avaliação de cada curso de ação depende de fatores concretos, como a gravidade da escassez alimentar, o nível de contaminação, a viabilidade técnica de mitigação e a distribuição social dos danos. A destruição protege melhor a saúde, mas pode aprofundar a privação alimentar. A diluição ou a transformação pode reduzir perdas em situações de urgência, embora levante dúvidas sérias quanto à segurança residual, à legalidade e à possibilidade de criar padrões duplos entre quem pode acessar alimentos seguros e quem acaba exposto ao que sobra. Os usos não alimentares afastam o risco humano direto, mas exigem condições técnicas e logísticas nem sempre disponíveis. Já o consumo sob risco talvez seja a alternativa menos defensável do ponto de vista normativo, embora continue a ser, em certas famílias, a resposta mais provável quando simplesmente não existe alternativa. Por isso, a decisão precisa ser contextualizada, explicada com transparência e acompanhada de incentivos institucionais capazes de desencorajar a ocultação de lotes contaminados e a negligência em relação às medidas preventivas.

## Ética de cada ação

O Quadro 3 resume as implicações normativas por ação.

**Quadro 3:** Análise de cada ação sob a perspectiva da ética normativa, de modo geral.

AÇÃO	PERSPECTIVA		
	DEONTOLÓGICA	VIRTUDE	CONSEQUENCIALISTA
Destruição	Cumpre normas de segurança ✓	Penaliza grupos vulneráveis e agrava a fome x	Protege a saúde a longo prazo, mas causa perdas imediatas ✓ x
Diluição ou transformação	Reduz contaminação; exige regulação ✓ x	Pode gerar padrões duplos e é pouco viável no nível familiar x	Aproveita lotes, mas mantém riscos residuais ✓ x
Usos não alimentares	Remove contaminante da dieta humana ✓	Recupera valor econômico, mas é pouco prático ✓ x	Evita o risco humano; transfere-o para animais ✓ x
Consumo sob risco	Viola normas e o direito à alimentação segura x	Inevitável para os desfavorecidos; revela falha estrutural ✓ x	Garante sobrevivência imediata, mas gera graves danos crônicos ✓ x

Fontes: Secretariado Técnico de Segurança Alimentar e Nutricional ^23^, Conselho de Ministros ^21^, Instituto Nacional de Normalização e Qualidade. ^33^, Partnership for Aflatoxin Control in Africa 6), Casadei 7, Southern African Development Community ^38^, Cardwell et al. ^37^, Ahlberg et al. ^34^, Tufts University et al. ^17^, African Union’s Interafrican Bureau for Animal Resources 5, Abbas et al. ^22^.

Nota: ✓ favorável; x desfavorável; ✓ x ambíguo.

A leitura comparada dessas três correntes mostra que elas não convergem plenamente. A deontologia tende a ser menos permissiva em relação ao consumo sob risco; o consequencialismo admite ponderações excepcionais diante da fome extrema; e a ética da virtude chama a atenção para a prudência, a compaixão, a justiça e a responsabilidade institucional. Essa divergência não enfraquece a análise. Ao contrário, ajuda a evidenciar a profundidade moral do dilema.

Destruição e uso não alimentar estão alinhadas ao enquadramento regulatório, mas enfrentam percepções sociais de “desperdício” e de baixo risco percebido (aflatoxinas são invisíveis e insípidas) ^24^ ^,^ ^28^ ^,^ ^33^ ^,^ ^34^.

Usos não alimentares exigem viabilidade técnica e logística comprovada (incluindo sanidade animal, quando aplicável). Automaticamente, ele não viola as leis relativas ao consumo humano. Se o desvio de aplicação for para o consumo animal, deve-se assegurar o cumprimento do Regulamento de Sanidade Animal, que prevê a apreensão e a interdição de forragens, rações e subprodutos destinados a animais que representem perigo à saúde dos animais ^35^. Se o alimento for destinado a outros fins, como a produção de biodiesel ou de álcool de laboratório, a lei moçambicana não o proíbe. Entretanto, em contextos de crise, um desvio tão radical de aplicação exige avaliação da viabilidade técnica e socioeconômica, pois requer uma capacidade técnica, tecnológica, logística e infraestrutural provavelmente inexistente.

## Solução proposta

Com base nos documentos analisados e na interpretação normativa aqui adotada, sustenta-se que não há solução moralmente isenta de custos em contextos de escassez extrema. A posição defendida neste ensaio não consiste em normalizar o consumo de alimentos contaminados, mas em reconhecer que, em situações-limite, a prioridade conferida à sobrevivência imediata pode tornar compreensíveis escolhas que, em condições ordinárias, seriam eticamente inaceitáveis. Ainda assim, isso não elimina o dever de reduzir o risco ao máximo. Nessas condições, a resposta mais defensável parece residir na redução máxima possível da exposição. Quando houver viabilidade técnica, a diluição ou a transformação com desvio para usos não alimentares pode funcionar como saída intermediária, desde que acompanhada de verificação laboratorial, comunicação clara do risco e supervisão institucional efetiva ^20^ ^,^ ^21^ ^,^ ^23^. Em paralelo, medidas preventivas, como boas práticas pós-colheita, biocontrole, armazenamento adequado e educação comunitária, continuam essenciais para evitar que o dilema se repita ^6^ ^,^ ^20^ ^,^ ^21^ ^,^ ^23^. Do ponto de vista ético, a resposta não deve consistir em deslocar silenciosamente o risco para os mais pobres, mas em criar condições para que essa escolha deixe de se impor. Isso exige decisões participativas e transparentes, alinhadas à Política e Estratégia de Segurança Alimentar e Nutricional (PESAN, em Moçambique, cobrindo 2024 a 2030), e maior articulação entre saúde pública, agricultura, regulação e proteção social ^21^ ^,^ ^22^ ^,^ ^23^ ^,^ ^36^.

## Conclusão

O dilema entre quantidade e qualidade alimentar não admite solução simples. A destruição protege a saúde, mas pode agravar a escassez; o consumo sob risco preserva a sobrevivência imediata, mas compromete o DHAA e expõe os mais vulneráveis a danos duradouros; a diluição, a transformação e os usos não alimentares surgem como respostas intermediárias, embora dependam de condições técnicas, normativas e institucionais nem sempre disponíveis.

Em contextos de fome extrema, as decisões assumem um caráter trágico porque obrigam a escolher entre danos concorrentes. Ainda assim, isso não elimina a responsabilidade pública. À luz da deontologia, da ética da virtude e do consequencialismo, a resposta mais defensável não é a normalização do risco, mas a construção de condições institucionais que impeçam as populações vulneráveis de terem de escolher entre comer e adoecer. Daí a urgência de reforçar políticas participativas, monitoria pós-colheita, tecnologias preventivas, capacidade analítica e comunicação de risco, a fim de reduzir a reincidência do problema.

## Data Availability

As fontes de informação utilizadas no estudo estão indicadas no corpo do artigo.
